# Memoir on the Odours Which Are Exhaled from the Body, Considered as Symptoms of Health and Disease

**Published:** 1799-05

**Authors:** C. Brieude


### *Memoir on the Odours which are exhaled from the Body, conſidered as ſymptoms of health and diſeaſe:—By* C. BRIEUDE.

*(Extracted from the 'Recueil periodique de la Societé de Médécine de Paris,' No. XXIX. Vol. V.)*

DAILY experience proves the importance of the olfactory ſenſations, and the neceſſity of analyſing them. The bad ſmells, which often afford indications to the phyſician, are moſt difficult to be deſcribed, and diſtinguiſhed accurately. In the ſame caſe where the relative chain of ideas would be entirely formed by habit, the difficulty of expreſſion would preſent a new obſtacle; and in fact, both ancient and modern languages are defective in terms to expreſs the different modifications of the olfactory perceptions; a certain proof, that notwithſtanding the number and frequency of theſe perceptions, a nomenclature of them has never been attempted.

The author has not treated the queſtion of odours in its full extent, but confined himſelf to thoſe which in man depend upon age, ſex, profeſſion, climate, diſeaſes, certain ſecretions, excretions, &c.

In children the predominance of acid ſmell is ſufficiently perceptible; it exhales from the cruſta lactea (*croûtes*), their ſweat, and moſt of their excrementitious evacuations: when it changes and approaches to an alkaline tendency, it indicates a diſpoſition to ſickneſs, as practitioners and nurſes know by experience. Several circumſtances may then produce a different modification of that ſmell peculiar to childhood.

In adults a ſtrong ſmell of a marked kind may be conſidered as a type, and taking the name of male odour. Luxury, and an exceſſive attention to the perſon, may diſguiſe it, without deſtroying it. This ſmell is neither inſupportable nor diſagreeable to women who know how to eſtimate it; and often produces in them, by the aſſociation of ideas, grief, deſire, hope, &c.

In

In reſpect of climate, light, and the action of caloric, increaſe the ſmell of human beings, as well as of the other productions of nature.

Aliment will, in like manner, influence the ſmell exhaling from man; and the oppoſite mode of living between the inhabitants of Quercy and thoſe of Upper Auvergne, has, in reſpect of ſmell, effects as different as the cauſes producing them.

Spirituous liquors, vegetable or animal diet, equally vary the ſmells which we exhale, and communicate to each individual a peculiar atmoſphere.

The ſmell of old age is ſimilar to that of childhood: it becomes inſipid and ſweetiſh in the cells of monks, in hoſpitals, and priſons.

The profeſſions which produce particular ſmells, are thoſe of butchers, tanners, candlemakers, and ſizers of paper.

Theſe obſervations on ſmell are inſufficient for the phyſician. It is alſo neceſſary for him to attend to all thoſe which depend on certain local diſpoſitions, or diſeaſes: febrile patients, phthiſical perſons, and women after child-birth, and certain kinds of ulcers, exhale particular odours. Brieude has not, perhaps, inſiſted ſufficiently on the febrile odour; on thoſe of putrid and malignant fevers; on that mouſe-ſmell *(odeur de ſouris)*, as the practitioners call it, which prevails in hoſpital and jail-fevers. From the gangrenous humid ulcers of hoſpitals, there ariſes alſo a very particular ſmell, which may be marked as a ſymptom. Cadaverous ſtools, their modifications in differrent epochs of a diſeaſe, ought in like manner to be attentively examined by the ſmell, and would thus become the ſource of very important medical indications.

